# Transparent semiconducting SrTiO_3_ crystal fabricated by heating treatment with gaseous ammonia and CeO_2_ powder

**DOI:** 10.1038/s41598-018-23019-9

**Published:** 2018-03-22

**Authors:** Yuka Morimoto, Junji Nishiyama, Hiroaki Takeda, Takaaki Tsurumi, Takuya Hoshina

**Affiliations:** Nano-phononics Lab., School of Materials and Chemical Technology, Tokyo Institute of Technology, Ookayama, Meguro, Tokyo, 152-8552 Japan

## Abstract

A transparent semiconducting SrTiO_3_ single crystal with a resistivity of the order of 10^3^ Ω·cm was fabricated by heating a SrTiO_3_ single crystal with gaseous ammonia and CeO_2_ powder. Conductive atomic force microscope (C-AFM) measurement revealed that micro-sized voids were formed and the high conductivity was exhibited only at around the voids. It is considered that the micro-sized voids were caused by the concentrated SrO planar defects, and TiO_2_-terminated structure with oxygen vacancies contributed to the two-dimensional conduction. In the heating process, the CeO_2_ powder acted as an oxygen source, and radicals such as NH_2_ and NH were generated by the reaction of oxygen and ammonia. The radicals may have contributed to the formation of three-dimensional network of the conductive paths consisting of SrO planar defects without the reduction of the bulk components. The electrons were localized on the TiO_2_-terminated structure, and the volume content of the conductive paths was small compared to the insulating bulk component. Therefore, the crystal was optically transparent and semiconducting.

## Introduction

Transparent conductive oxides are widely used in optoelectronic applications such as solar cells, UV-light emitting diodes (LEDs), transparent thin film transistors (TFTs), photochromic devices, gas sensors, and infrared absorbing materials^1–3^. Generally high optical transparency is incompatible with high electronic conduction, since optical transparency requires a band gap of 3.3 eV or more and such a large gap makes carrier doping difficult^1^. In this sense, transparent conductive oxides are exceptional materials. As typical alternatives, In_2_O_3_:Sn, SnO_2_:Sb, SnO_2_:F, ZnO:Al, ZnO:Ga and TiO_2_:Nb are well known^2,3^. These transparent oxide semiconductors are based on the creation of electron degeneracy in wide band gap oxides either by substitution of some of the atoms or by oxygen vacancies^2,3^. On the other hand, different type of the transparent conductive oxide materials based on low-dimensional transportation have also been reported^4,5^. For example, the (100) surface of SrTiO_3_ with SrO- or TiO_2_-terminated structure exhibits metallic states when electron is created on the surface by oxygen vacancies or hydrogen adsorption^4–7^. In these materials, the energy level of the surface state is formed in the band gap of the underlying bulk crystal and the Fermi level is in the conduction band^8^. Since the electron mobility is high and the thickness of space-charge layer is several nanometers, high conductivity can be obtained even if the crystal looks optically transparent.

We think that the low-dimensional transportation occurs also on the interface of stacking fault formed in an insulating crystal. Recently, Shkabko and co-workers have reported nitrogen-doped strontium titanate crystals fabricated by microwave induced plasma nitridation of SrTiO_3_^9–15^. The nitrogen-doped strontium titanate crystals have stacking faults originates from vacancies of strontium and oxygen atoms, and the stacking faults exhibit high electrical conductivity. However, the mechanisms of the electrical conduction and the formation of the stacking faults are not clear yet.

In contrast to their study, we have reported that the nitrogen-doped strontium titanate single crystal heated in gaseous ammonia was an excellent dielectric^16^. Our result is contrary to the Shakabko’s result although both of the samples have been fabricated by heating treatment of SrTiO_3_ single crystal with gaseous ammonia. At a high temperature, ammonia decomposes to nitrogen and hydrogen, and radicals such as NH_2_* and NH* are generated as intermediates^17^. These chemical species take part in a reduction reaction as well as a nitriding reaction of strontium titanate. The reduction reaction causes generation of oxygen vacancies providing N-type conductivity. In contrast, nitrogen acts as an acceptor. The sufficiently nitrided strontium titanate has the chemical composition of SrTiO_3−3*x*_N_2*x*_, in which the charge balance is maintained, and represents an insulation property. Accordingly, it is possible that the chemical stability of NH_2_ and NH radicals in the heating process affects the electrical property of strontium titanate.

It has been reported that the amount of the NH_2_ and NH radicals increases when oxygen gas is mixed into ammonia gas at a high temperature^18,19^. Therefore, the amount of the radicals may be controlled by providing oxygen gas during the heating treatment with ammonia gas. However, the reaction of oxygen and ammonia is danger because of the explosiveness of ammonia. In addition, the radicals might have to be generated near SrTiO_3_ crystal to react with the crystal because of the short lifetime of radicals. CeO_2_ is a well-known substance which generates oxygen gas changing the valance of Ce according to the following equation at a temperature higher than 900 K^20,21^.

1 $${{\rm{C}}{\rm{e}}{\rm{O}}}_{2}\to {{\rm{C}}{\rm{e}}{\rm{O}}}_{2-{\rm{x}}}+\frac{{\rm{x}}}{2}{{\rm{O}}}_{2}$$

Therefore, oxygen gas can be provided near the sample without the risk of explosion when a small amount of CeO_2_ powder was set close to the sample. Accordingly, we used CeO_2_ powder as an oxygen source in a heating treatment of SrTiO_3_ single crystal with gaseous ammonia to control the amount of NH_2_ and NH radicals in this study. As the result, a transparent semiconducting SrTiO_3_ single crystal was obtained. We introduce the fabrication process of the crystal and the mechanism of transparent conductivity in this article.

## Results and Discussion

**Figure 1:**
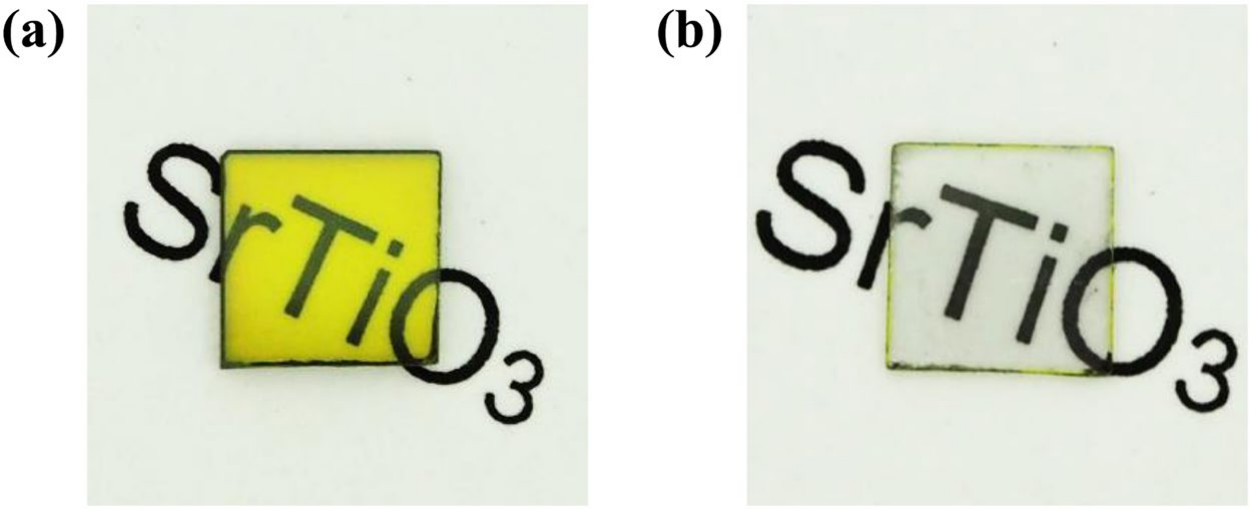
(**a**) Photograph of the SrTiO_3_ single crystal heated with CeO_2_ powder in gaseous ammonia. The surface color of the crystal is light yellow, suggesting the surface layer was nitrided. (**b**) Photograph of the inside part of the crystal heated with CeO_2_ powder in gaseous ammonia. The sample changed gradually from yellow to colorless with decreasing the sample thickness, and the sample changed to totally transparent when the polishing amount was approximately 160 µm (total on both sides).

**Figure 2:**
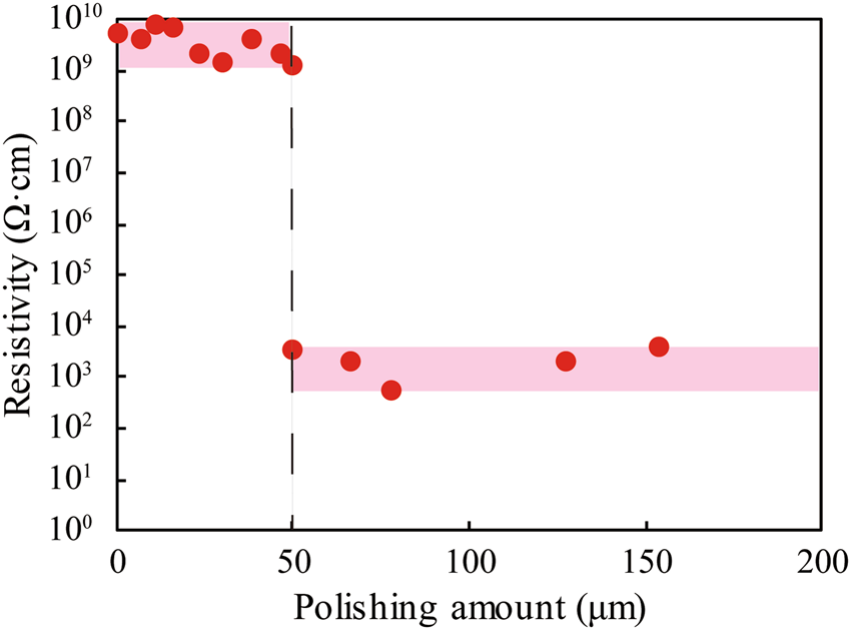
Resistivity of the SrTiO_3_ crystal heated with gaseous ammonia and CeO_2_ powder as a function of the polishing amount of the both surfaces. While the resistivity was on the order of 10^9^ Ω·cm before the polishing, the resistivity drastically dropped to 3.3 × 10^3^ Ω·cm when the polishing amount was 50 µm. The resistivity was on the order between 10^2^ and 10^3^ Ω·cm for the larger polishing amount.

(100)-oriented SrTiO_3_ single crystal with a size of 5 × 5 × 0.5 mm^3^ was heated with CeO_2_ powder in gaseous ammonia at 1000 °C for 15 h using a tubular electric furnace. Figure 1(a) shows a photograph of the obtained single crystal. The surface color of the as-prepared crystal was light yellow, suggesting that the surface layer was nitrided. The bandgap of nitrogen-doped strontium titanate is generally narrower than pure SrTiO_3_, and therefore, SrTiO_3_ changed from colorless to yellow due to the substitution of oxygen by nitrogen^16,22^. The apparent relative permittivity at 1 MHz was 1,500 (Supplementary Information, Fig. S1(a)), which was significantly larger than that of the pure SrTiO_3_, 310^16^. The Cole-Cole plot of the crystal showed multiple circular arcs, suggesting a structural nonuniformity of the crystal (Supplementary Information, Fig. S1(b)). The large apparent permittivity was due to interfacial polarizations. Therefore, we polished both the upper and lower surfaces of the crystal, and measured the bulk resistivity after forming In-Ga electrodes on both sides. Figure 2 shows the transition of the resistivity as a function of the polishing amount of the both surfaces. While the resistivity was on the order of 10^9^ Ω·cm before the polishing, the resistivity drastically dropped to 3.3 × 10^3^ Ω·cm when the polishing amount was 50 µm. The resistivity was on the order between 10^2^ and 10^3^ Ω·cm for the larger polishing amount. In addition, it was confirmed that the inside part of crystal was a N-type semiconductor by detecting a thermal electromotive force due to the Seebeck effect. These result suggested that both the upper and lower surfaces of the crystal were insulating and the inside part exhibited a semiconducting property. Figure 1(b) shows a photograph of the crystal polished until the thickness of the crystal became 340 μm. The sample changed gradually from yellow to colorless with decreasing the sample thickness, and the sample changed to totally transparent when the polishing amount was approximately 160 µm (total on both sides).

**Figure 3:**
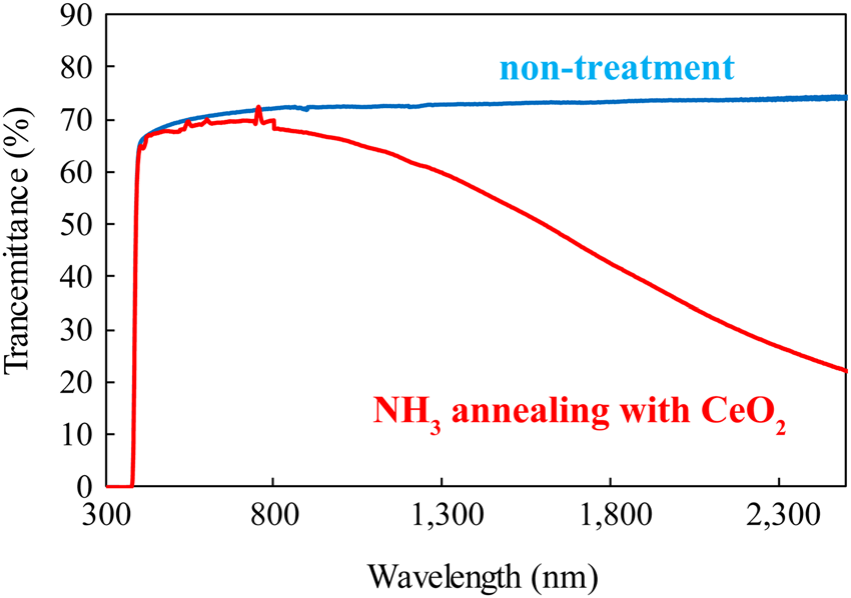
Transmittance spectra of the semiconducting inside part of single crystal heated with CeO_2_ powder in gaseous ammonia and as-purchased SrTiO_3_ single crystal.

Figure 3 shows the transmittance spectra of the semiconducting inside part and the as-purchased SrTiO_3_ single crystal measured by an ultraviolet-visible (UV-Vis) and infrared (IR) spectrometers. First, the absorption edge of the semiconducting inside part was exactly same as the as-purchased SrTiO_3_ single crystal and the transmittance was about 70% in the visible light region. This meant that the band gap of the semiconducting inside part did not change by NH_3_ heating treatment with CeO_2_ powder, and oxygen was not substituted by nitrogen in the inside part of crystal. As shown in Fig. S2 in the Supplementary Information, the absorption edge of the whole part of crystal seems to be shifted from 380 to 420 nm, which indicates that the only the surface of crystal was nitrided by NH_3_ heating treatment with CeO_2_ powder. (The energy level of N 2p orbitals in N-doped SrTiO_3_ is above that of O 2p orbitals, and the mixed orbitals of N 2p and O 2p compose the valence band, whereas the valence band for pure SrTiO_3_ consists of O 2p orbitals. Therefore, the band gap of N-doped SrTiO_3_ should be narrower than that of pure SrTiO_3_)^16,22^. In addition, nitrogen in perovskite structure was not detected in the inside part of crystal even by X-ray photoelectron spectroscopy (XPS). From these results, it was found that the transparent semiconducting inside part was not nitrided, while the yellow insulating surface layer was a nitrided layer. In the transmittance spectrum of the semiconducting inside part shown in Fig. 3, absorption attributed to Ti^3+^ at around 520 nm was not observed. Typical N-type semiconductor of SrTiO_3_ has reduced Ti^3+^, and then the color of the semiconducting SrTiO_3_ is generally dark-blue^23,24^. Nevertheless, our semiconductive sample did not show the Ti^3+^ absorption. On the other hand, absorption due to the plasma oscillation was observed in the IR wavelength region. This meant that the sample had free electrons and high conductivity. Therefore, the optical property indicated that the inside part was completely transparent despite being a semiconductor.

To verify the effects of CeO_2_ powder, a SrTiO_3_ crystal was heated without CeO_2_ powder in gaseous ammonia. The color of the sample heated without CeO_2_ powder was deep yellow (Supplementary Information, Fig. S3), and the relative permittivity at 1 MHz was 310, which was almost the same as that of pure SrTiO_3_ (Supplementary Information, Fig. S4(a)). The Cole-Cole plot showed a single arc, suggesting that the sample heated without CeO_2_ powder had homogeneous structure (Supplementary Information, Fig. S4(b)). As shown in Fig. S5 (Supplementary Information), the resistivity of the sample heated without CeO_2_ powder was higher than that of the non-annealed crystal, and remained at a high value of 10^9^–10^11^ Ω·cm even after polishing. The color of the sample hardly changed before and after polishing. Figure S2 (Supplementary Information) shows the transmittance spectrum of the sample heated without CeO_2_ powder. The absorption edge was approximately 460 nm, suggesting that the sample heated without CeO_2_ powder was more nitrided than the sample heated with CeO_2_ powder. In addition, the absorption due to plasma oscillation was not observed in the transmittance spectrum. These results indicated that the sample heated without CeO_2_ powder was totally nitrided, and the electron carrier concentration was extremely low compared to the sample heated with CeO_2_ powder. It is because doped nitrogen acts an acceptor, and a fully nitrided sample should exhibit insulation.

**Figure 4:**
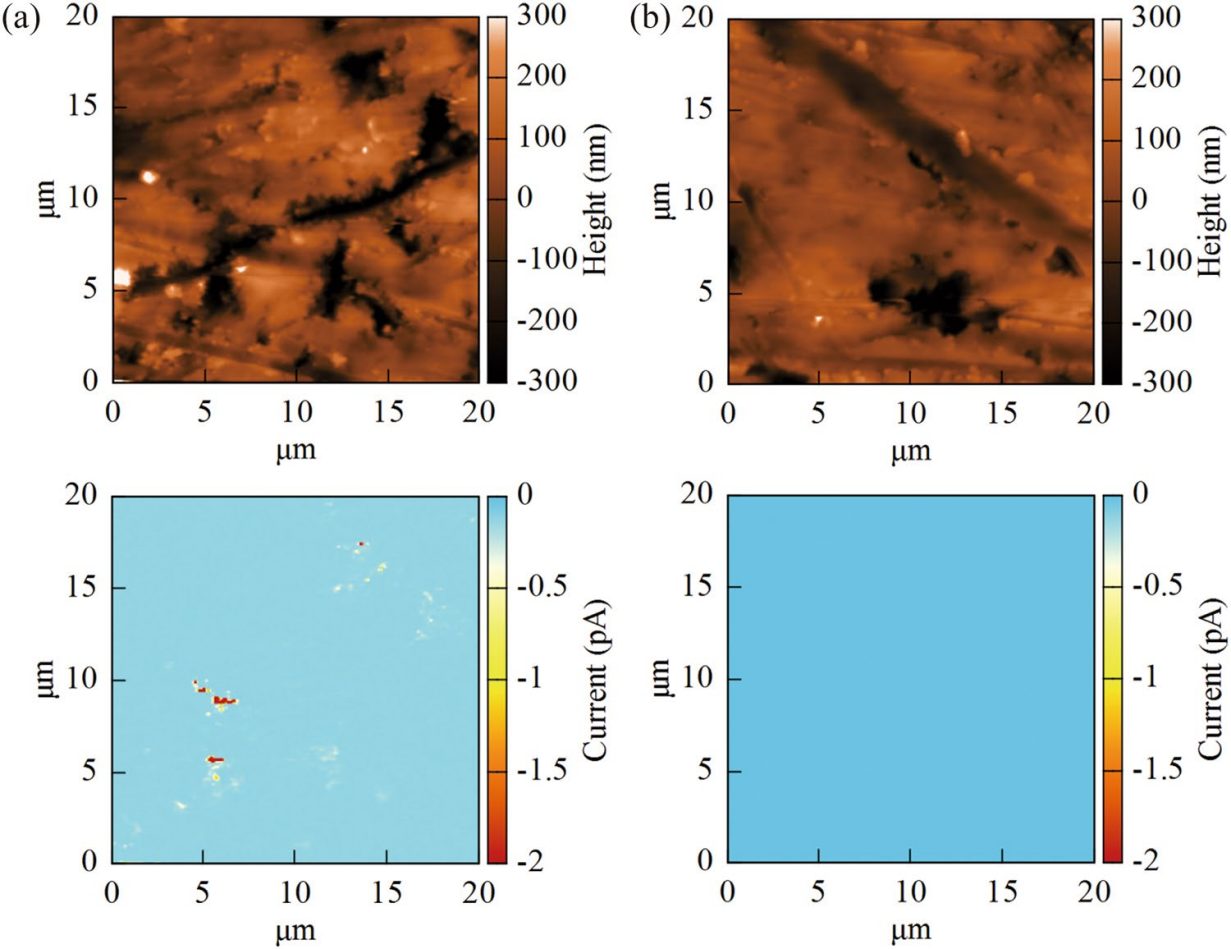
Surface topography and current distributions of semiconducting inside part (**a**) and surface nitrided layer (**b**) of the crystal heated with NH_3_ and CeO_2_ powder (C-AFM images). The upper and lower figures indicate mapping profiles of height and current, respectively. Micro-sized voids were formed on both of the surface, however, the current was detected only at deep voids on the surface of the transparent inside part.

**Figure 5:**
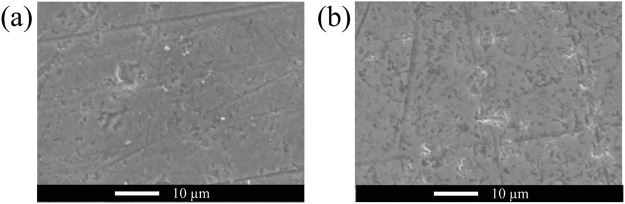
SEM images of the surface of the semiconductive inside part (**a**) and surface nitrided layer (**b**) after polishing. The number density of micro-sized voids in the inside part was lower than that in the surface nitrided layer.

To understand the mechanism of the high conductivity measured for the inside part of the crystal heated with NH_3_ and CeO_2_ powder, we measured the topography and the current distribution over the sample surface using a C-AFM. As shown in Fig. 4(a), micro-sized voids were formed on the polished sample and large currents were detected only at the deep voids. This result suggested that there were nano-scale conductive paths around the corn-shaped voids while the bulk component was insulating. It was reasonable that the sample looked optically transparent since the major volume part consisted of an insulating bulk strontium titanate. On the other hand, micro-sized voids also existed on the surface nitrated layer, however, conductive path was not present in nitrided layer, as shown in Fig. 4(b). Figure 5 shows surface morphology of the semiconductive inside part and surface nitrided layer after polishing. There are many micro-sized voids on either surface, and therefore the micro-sized voids may have formed by the polishing treatment. However, conduction paths were present only in the inside part of crystal heated with NH_3_ and CeO_2_. It is noted that number density of micro-sized voids in the inside part was lower than that in the surface nitrided layer.

**Figure 6:**
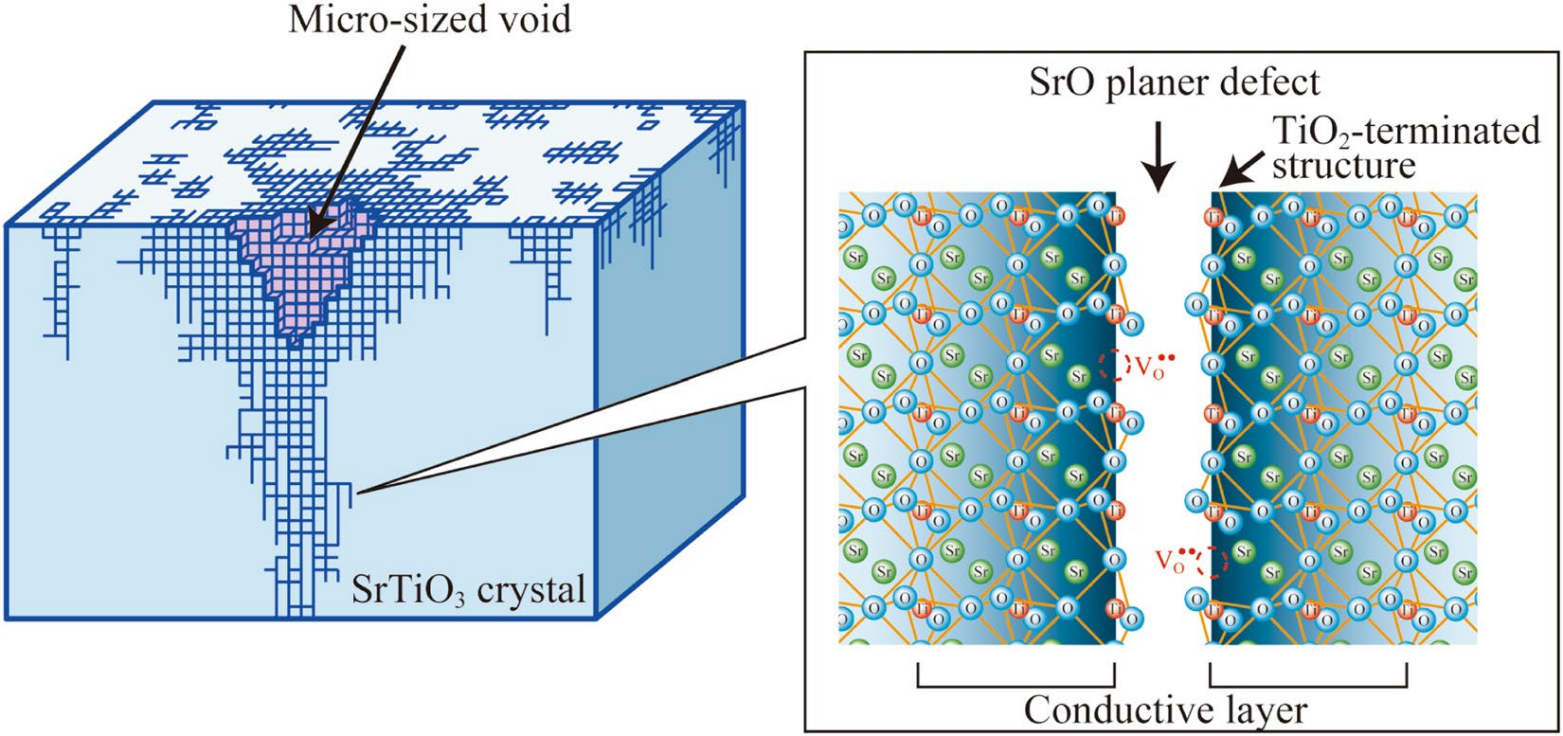
Schematic illustration of a micro-sized void and conductive paths of inside part of the crystal heated with NH_3_ and CeO_2_ powder. It is considered the micro-sized voids were caused by the concentrated SrO planar defects, and TiO_2_-terminated structure with oxygen vacancies contributed to the two-dimensional conduction.

We discuss the reason why conduction paths were present only in the inside part of crystal heated with NH_3_ and CeO_2_. It has been reported that stacking faults consisted of SrO planar defects are easily formed in a SrTiO_3_ single crystal annealed in a reducing atmosphere^10,15,25^. In other words, TiO_2_-terminated structures are exposed on the surface of the defect parts. In our experiment, micro-sized voids were observed by the SEM and C-AFM. It is considered that oxygen vacancies were introduced by the reduction reaction with NH_3_, NH_2_*, NH*, and H_2_. The strontium atoms evaporated or moved to sample surface with the formation of oxygen vacancies, resulting the formation of SrO planar defects^10,15,25^. Therefore, it is reasonable to think that the micro-sized voids were related to the stacking faults consisted of SrO planar defects. Since the part where the stacking fault concentrated was mechanically weak, the micro-sized voids were prone to be formed at the stacking-fault-concentrated part by polishing, as shown in Fig. 6. In addition, it is known that TiO_2_-terminated surface of (100)-oriented SrTiO_3_ single crystal has a metallic state when oxygen vacancies exist on the surface^4,6^. The space-charge layer is formed near the surface, and two-dimensional electron transportation occurs in a region located at the depth of several nanometers from the surface, which is nearly equal to the Debye length^5^. Also in the case of the inside part of the crystal heated with NH_3_ and CeO_2_, it is considered that a similar TiO_2_-terminated structures, in which the concentration of oxygen vacancies was high to some extent, were formed around the SrO planar defects, and they contributed to the two-dimensional conduction. Since the concentration of the planar defects was higher near the micro-sized voids, the high conductivity was obtained only at around the micro-sized voids. To confirm whether the conduction related to the surface state, we measured the resistivity of the sample immediately after re-annealing at 500 °C with nitrogen. The resistivity dropped from the order of 10^3^ Ω·cm to 1.8 × 10^0^ Ω·cm (Supplementary Information, Fig. S6), suggesting the formation of oxygen vacancies on the TiO_2_-terminated structure or the desorption of the surface-adsorbed-substances such as hydroxyl groups (−OH), i.e., the conduction was affected by the surface state evidently. Since the conductivity did not highly depend on the sample thickness when the thickness was 340 μm or less, it is thought that the SrO planar defects existed uniformly and formed a three-dimensional network of conductive paths in the inside part of crystal. It is thought that the SrO planar defects also existed in the nitrided surface layer before polishing. However, the surface layer was insulating since electron carriers were cancelled due to the presence of nitrogen acting as an acceptor.

When the electrons were localized on the TiO_2_-terminated structure, there is no necessity that the underlying bulk crystal becomes colored. In spite of it, previous studies have reported that SrTiO_3_ crystals having conductive paths obtained by a heating treatment in a reduction atmosphere is dark-blue-colored unlike our crystal^23,24^. The reduced crystals have oxygen vacancies in the crystal lattices, and the dark-blue color is due to the reduction of titanium from Ti^4+^ to Ti^3+^ for the charge compensation of the oxygen vacancies in the bulk. In the case of our crystal, conductive paths were efficiently formed without the reduction of the bulk component. Therefore, the crystal showed transparent conductivity. The reaction mechanism is not clear, however, radicals such as NH_2_* and NH* should have contributed to the formation of the conductive SrO planar defects without the reduction of the bulk component. The concentration of the SrO planar defects can be controlled by heating treatment with gaseous ammonia and CeO_2_ powder.

In this study, transparent and semiconducting SrTiO_3_ bulk crystal was obtained by heating treatment with NH_3_ and CeO_2_. The conduction mechanism of our crystal is different from that of general transparent oxide semiconductors. General transparent oxide semiconductors are based on the creation of electron degeneracy in wide band oxides either by substitution of some of the atoms or by oxygen vacancies^2,3^. In contrast, our transparent semiconducting crystal was realized by low-dimensional transportation on defect structure. Although the value of the conductivity was considerably lower than that of the existing transparent semiconductors, the conductivity may be increased by increasing conduction path. Since SrTiO_3_ is the most common material in perovskite-type oxides, the new finding on low-dimensional transportation may contribute to fundamental solid state physics. In addition, the transparent semiconducting SrTiO_3_ crystal could be used as a transparent semiconductive substrate for optical devices using perovskite-type functional materials. In the present situation, it may be difficult to obtain sufficient function as an electrode only with the transparent semiconducting SrTiO_3_ crystal. It would be effective to deposit an ultrathin film of Pt or SrRuO_3_ on the transparent semiconducting SrTiO_3_ crystal for functioning as a transparent electrode^3^. In addition, application to infrared absorbing materials can also be expected.

In summary, a SrTiO_3_ single crystal was heated with CeO_2_ powder in gaseous ammonia. The CeO_2_ powder acted as an oxygen supply source, and contributed to the generation of radicals such as NH_2_ and NH. The surface layer of the obtained crystal was yellow-colored insulator, meaning that the surface layer was nitrided. In contrast, the inside part of crystal was transparent semiconductor with a resistivity of 10^3^ Ω·cm order. The result of the C-AFM measurement revealed that micro-sized voids existed on the polished surface and the conductivity was exhibited only at around the voids. Although the micro-sized voids were due to polishing treatment, the micro-sized voids were related to the stacking faults consisted of SrO planar defects. Since the part where the stacking fault concentrated was mechanically weak, the micro-sized voids were prone to be formed at the stacking-fault-concentrated part by polishing. It was thought that the SrO planar defects with oxygen vacancies contributed to the two-dimensional conduction, and a three-dimensional network of conductive paths was formed in the inside part of crystal. By the heating treatment with NH_3_ and CeO_2_, the two-dimensional conductive paths were efficiently formed without the reduction of the bulk component, and therefore, the transparent conductive crystal was obtained.

## Method

(100)-oriented SrTiO_3_ single crystal purchased from Shinkosha was used as a raw material. The size of crystal was 5 × 5 × 0.5 mm^3^, and both surfaces of crystal were polished to a mirror finish. The single crystal was heated with or without CeO_2_ powder in gaseous ammonia at 1000 °C for 15 h using a tubular electric furnace (Supplementary Information, Fig. S7(a)). The single crystal was set at the center of the furnace, and 0.0075 mol of CeO_2_ powder (oxygen source) was heaped up on the both sides (Supplementary Information, Fig. S7(b)). The flow rate of gaseous ammonia was controlled at 0.1 L/min using a mass flow controller.

The dielectric property was measured by an impedance analyzer (Agilent, 4294A) after forming In-Ga electrodes on the both surface. The resistivity of the crystal was measured by an ultra high resistance meter (ADCMT, 8340A) at room temperature with a source bias of 1 V. The topography and current distribution were measured using a SEM (JEOL, JCM-6000Plus) and C-AFM (Asylum Research, Cypher). The tip of the cantilever for the C-AFM measurement was made of Ti/Ir-coated Si with a radius of 28 ± 10 nm (ASYELEC.01-R2). The applied voltage was −8 V.

## Electronic supplementary material

Supplementary information
