# Supplementary material for: Transparent semiconducting SrTiO3 crystal fabricated by heating treatment with gaseous ammonia and CeO2 powder
Source: Sci Rep. 2018 Mar 22;8:5031. doi: 10.1038/s41598-018-23019-9 (PMC5864887; doi:10.1038/s41598-018-23019-9)
Supplement: Supplementary file 1 — Supplementary information [file 41598_2018_23019_MOESM1_ESM.pdf]

Supplementary Information for

Transparent semiconducting $SrTiO_3$ crystal fabricated by heating treatment with gaseous ammonia and CeO_2 powder

Yuka Morimoto, Junji Nishiyama, Hiroaki Takeda, Takaaki Tsurumi, and Takuya Hoshina*

Nano-phononics Lab., School of Materials and Chemical Technology, Tokyo Institute of Technology, Ookayama, Meguro, Tokyo 152-8552, Japan

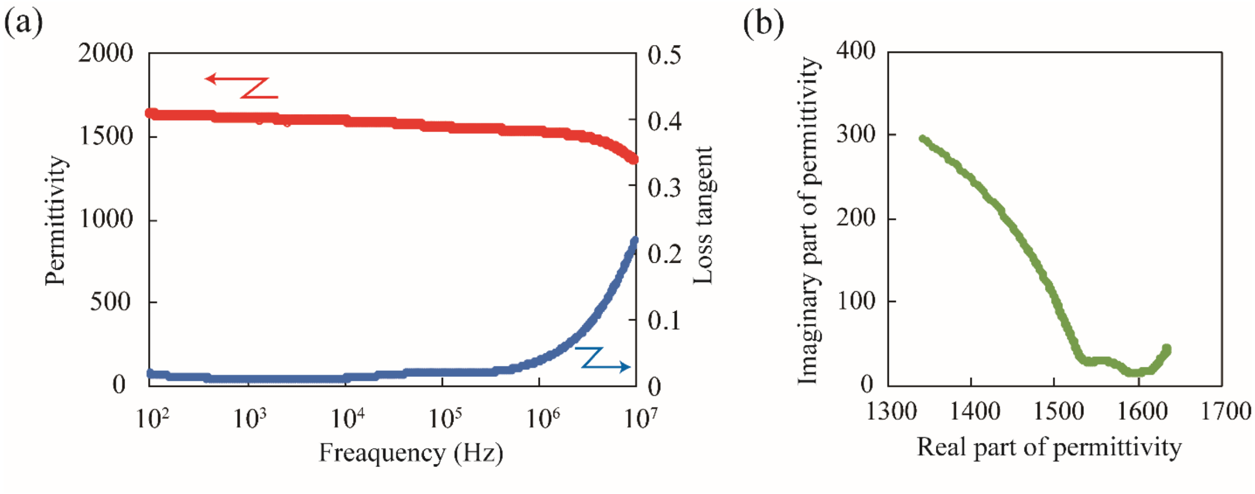

Figure S1. (a) Dielectric property and (b) Cole-Cole plot of the crystal annealed with CeO_2 powder in gaseous ammonia. The relative permittivity (ε_r = 1500 at 1 MHz) of the obtained crystal was larger than that of the pure $SrTiO_3$ (ε_r = 310). Cole-Cole plot indicates several circular arcs, suggesting that the large apparent permittivity was due to an interfacial polarization.

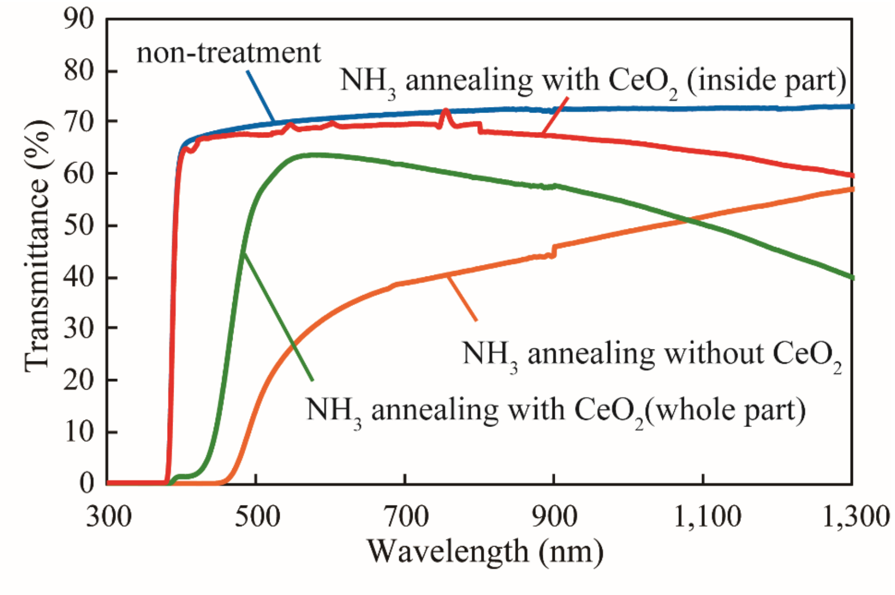

Figure S2. Transmittance spectra of the whole and inside parts of the crystal annealed with CeO_2 powder in gaseous ammonia, whole part of the crystal annealed without CeO_2 powder in gaseous ammonia and the as-purchased $SrTiO_3$ single crystal measured by an ultraviolet-visible (UV-Vis) and infrared (IR) spectrometers. The absorption edge of the whole part of crystal annealed with CeO_2 powder seems to be shifted from 380 to 420 nm. It suggests that the surface of the crystal was nitrided while the inside part of the crystal was not nitrided. On the other hand, the absorption edge of the crystal annealed without CeO_2 powder was also shifted by nitridation, however, the absorption due to the plasma oscillation was not observed, and therefore the electron carrier concentration was extremely low compared to that of the sample annealed with CeO_2 powder.

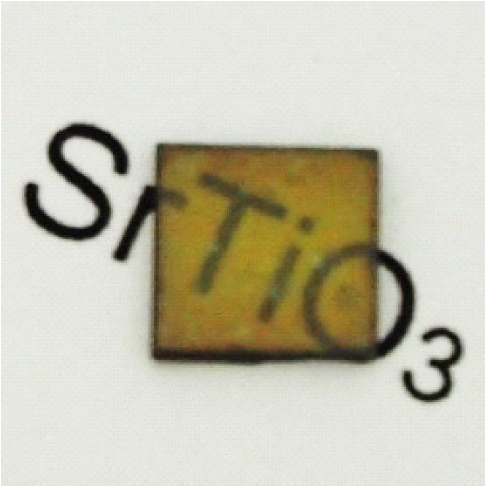

Figure S3. (a) Photograph of the $SrTiO_3$ single crystal heated without CeO_2 powder in gaseous ammonia. The color of the crystal was deep yellow, suggesting that the crystal was nitrided.

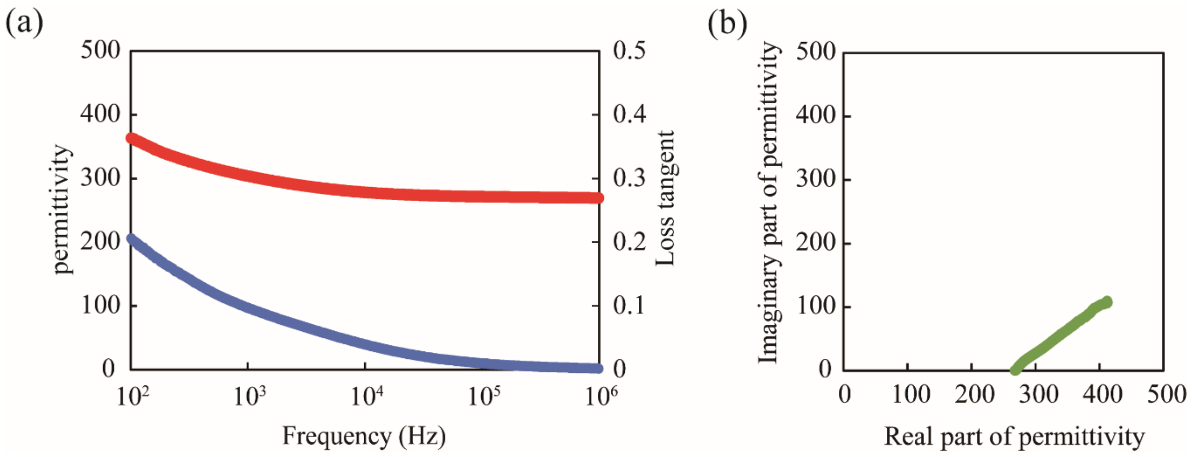

Figure S4. (a) Dielectric property and (b) Cole-Cole plot of the crystal annealed without CeO_2 powder in gaseous ammonia. The relative permittivity was same as that of the pure $SrTiO_3$ (ε_r = 310). Cole-Cole plot indicates a single arc, suggesting that the structure had a homogeneous structure.

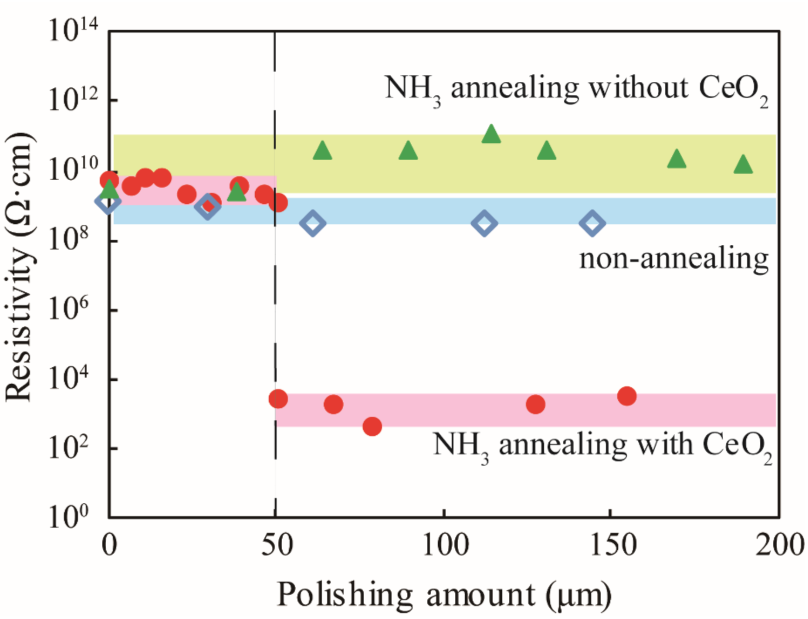

Figure S5. Resistivity of the crystal annealed with or without CeO_2 powder in gaseous ammonia, and non-annealed crystal as a function of the polishing amount of the both surfaces.

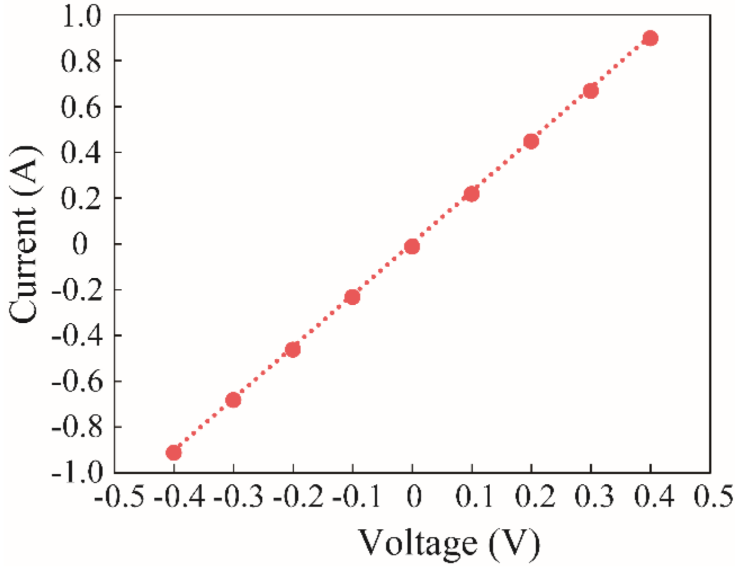

Figure S6. Current–voltage characteristic of the sample annealed in nitrogen at 500 °C after the heating treatment with gaseous ammonia and CeO_2 powder, and polishing. The sample showed ohmic characteristics, and the resistivity was estimated as 1.8×10^0 Ω·cm.

(a)

(b)

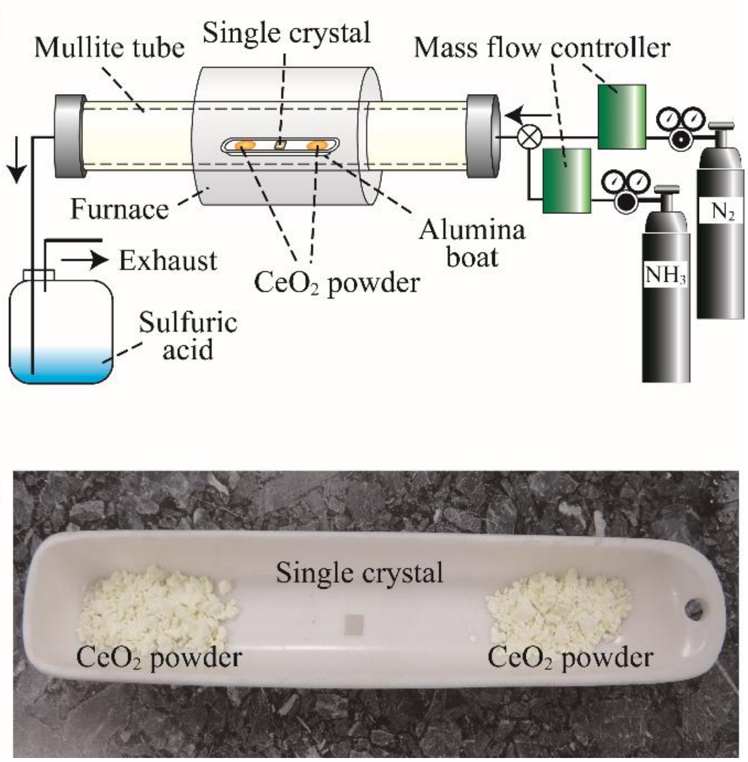

Figure S7. (a) Schematic illustration of the equipment for the heating treatment. Ammonia gas was provided from the edge of the mullite tube with a length of 1 m and a diameter of 5 cm. The nitrogen gas was used for inert gas replacement. The gases were exhausted after passing thorough sulfuric acid. (b) Photograph of the crystal before ammonia annealing with CeO_2 powder. The single crystal was set at the center of the alumina boat with a length of 15.5 cm and a width of 3 cm, and 0.0075 mol of CeO_2 powder (oxygen source) was heaped up on the both sides.
